# Employability Paradox: The Effect of Development Idiosyncratic Deals on Recipient Employees’ Turnover Intention

**DOI:** 10.3389/fpsyg.2021.696309

**Published:** 2021-08-04

**Authors:** Xiaoyan Zhang, Hui Deng, Yuhuan Xia, Yuanyuan Lan

**Affiliations:** School of Economics and Management, Beijing Jiaotong University, Beijing, China

**Keywords:** development idiosyncratic deals, internal employability, external employability, turnover intention, opportunity to perform

## Abstract

Applied social cognitive theory, this study built a moderated mediation model to explain how and when development idiosyncratic deals (i-deals) affect recipients’ turnover intention. Specifically, this study proposed two paths that linked development i-deals with the recipients’ turnover intention. One path was a retention path via perceived internal employability and another path was a turnover path via perceived external employability. This study tested the hypotheses with a sample of 337 employees from three companies in China. The results showed that development i-deals improved recipients’ perception of internal and external employability both. Perceived internal employability predicted low risk of turnover, but perceived external employability predicted high risk of turnover. And perceived internal and external employability played mediating roles in the relationship between development i-deals and turnover intention. Furthermore, the recipients’ perception of opportunity to perform in current organization strengthened the relationship between perceived internal employability and turnover intention, but weakened the relationship between perceived external employability and turnover intention. Theoretical and practical implications of these findings were discussed.

## Introduction

Idiosyncratic deals (i-deals) are voluntary and personalized agreements of a non-standard nature negotiated between individual employees and employers regarding employment terms that can benefit each party (Rousseau et al., 2006). Development i-deals refer to the individualized opportunities to develop working skills, enhance professional competencies, and meet personal career aspirations, which are essential for higher performance, greater occupational success, and a larger space for promotion (Zhang et al., 2020). In human resource management practice, development i-deals often manifest as challenging work assignments, special training opportunities, or career development opportunities in the organization (Marescaux et al., 2019; Zhang et al., 2020).

As an effective human resources management tool, development i-deals have been shown to have significant effects on recipient employees (Hornung et al., 2010, 2011). The majority of existing studies have tended to focus on the positive effects of development i-deals on the recipients’ work-related behaviors, such as constructive voice behavior (Ng and Feldman, 2015), helping behavior (Guerrero and Challiol-Jeanblanc, 2016), creativity (Wang et al., 2018), in-role performance, and organizational citizenship behavior (Anand et al., 2010, 2018). Only a few studies have noted that these i-deals can also exert some negative impacts on the recipients. For example, current research has indicated that development i-deals can increase recipients’ working stress and work-family conflicts (Hornung et al., 2014), feeling of being envied (2017), and even unethical pro-organizational behavior (Jiang and Zhang, 2020). However, thus far, empirical research on the potential costs and risks of granting development i-deals still remains in its infancy.

Development i-deals were originally used for retaining the employees that supervisors valued (Rousseau et al., 2006). Indeed, empirical research has shown that i-deals are effective in boosting recipients’ job motivation and commitment to the current organization (Bal et al., 2012). However, there is a common phenomenon that many recipient employees who are valued by their supervisors leave their organizations after obtaining special career development or training opportunities. To date, the research examining how and when development i-deals shape the recipients’ turnover intention is relatively rare (Brzykcy et al., 2019). Exploring the process through which development i-deals impact recipients’ turnover intention has important significance (Ng, 2017). Authorizing development i-deals to particular employees not only means that supervisors provide training and career development opportunities to those employees, but also means that the organization invests substantial direct and indirect money and time on the recipient employees (Rousseau et al., 2006). Therefore, once the recipients leave, it will result in costs to the organization. Thus, to advance the understanding of *whether, how, and when* development i-deals influence recipients’ turnover intention, a deeper investigation is warranted.

Accordingly, this study presents a framework based on social cognitive theory to reveal the cognitive and psychological mechanisms through which development i-deals may affect recipients’ turnover intention. The social cognitive theory posits that the development of individuals’ self-efficacy may be a conduit between environmental cues and their behavioral outcomes (Bandura, 1986). Obtaining development i-deals can be regarded as a valuable environmental cue because it provides desired resources to the recipients; thus, it will be more likely to boost their self-efficacy (Wang et al., 2018). Scholars have indicated that the conception of perceived employability is a form of self-efficacy (Nelissen et al., 2017). Rothwell and Arnold (2007) stated that perceived employability emphasizes the extent to which individuals believe that they have the ability to meet the requirements of the job they have (i.e., perceived internal employability) or the extent to which the individuals believe that they have the capability to attain the requirements of a certain new job that they desire (i.e., perceived external employability). Thus, this paper proposed that recipients’ cognition of their working capability may serve as a mediating variable, such that development i-deals may have positive effects on recipients’ perceived internal and external employability. Subsequently, this paper speculated that recipients’ improved perception of internal employability may increase recipients’ retention, which brings benefits to the organization, but the perception of external employability may increase recipients’ turnover, which brings some costs to the organization. Consistent with previous research, such a dilemma between the benefits and costs of recipients’ development i-deals can be referred to as the employability paradox (Nelissen et al., 2017).

Further, this study examined the moderating effect of recipients’ perception of opportunity to perform in current organization on the relationship between perceived employability and turnover intention. The opportunity to perform refers to the extent to which individuals believe they have sufficient opportunities to demonstrate their abilities, skills, and knowledge in the current organization (Schleicher et al., 2006; Ingold et al., 2016). Existing research has emphasized the importance of opportunity to perform, indicating that lack of opportunity to perform is related to performance decrements (Ford et al., 2006), which may affect employees’ willingness to remain. Thus, this study included opportunity to perform as a moderator in the model, and argued that high level of opportunity to perform may strengthen the negative relationship between perceived internal employability and turnover intention, but weaken the positive relationship between perceived external employability and turnover intention.

**FIGURE 1:**
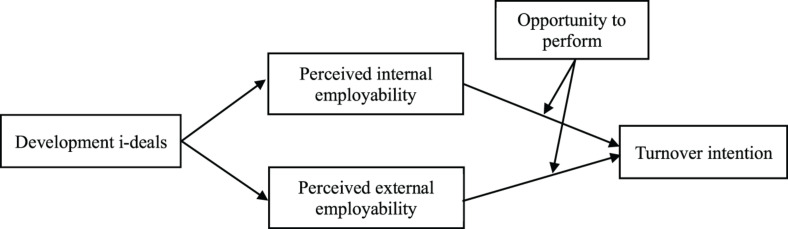
Theoretical model.

In sum, this paper applied social cognitive theory to explore the relationship between development i-deals and recipient employees’ turnover intention by uncovering the potential mediating cognitive mechanism of employability paradox and moderating effect of opportunity to perform. This provides a new theoretical perspective to understand the relationship between development i-deals and recipients’ behaviors. Figure 1 shows the conceptual model.

## Literature Review and Hypotheses Development

I-deals may take many forms in management practice (Rousseau et al., 2006). In terms of the content, scholars identified three types of i-deals, namely development i-deals (or task i-deals), flexibility i-deals, and reduced workload i-deals (Rousseau and Kim, 2006; Hornung et al., 2009). Based on this, Rosen et al. (2013) modified and broadened the previous scale of i-deals to cover four dimensions, namely schedule flexibility i-deals, location flexibility i-deals, task and work i-deals, and financial incentives i-deals. This study mainly focused on development i-deals. That is because, conceptually, development i-deals refer to the customized but limited opportunities in the organization that can increase the recipients’ working skills or promote their career development, while flexibility i-deals provide recipients with discretion to personalize working schedules or working locations to better fit personal needs (Hornung et al., 2014). Reduced workload i-deals are used to individually adjust the quantity or quality of workload, such as shorter workdays or less strenuous tasks (Hornung et al., 2009), and financial incentives i-deals allow employees to negotiate the terms of their compensation plans (Rosen et al., 2013). From the perspective of involved content, relative to other three types of i-deals, development i-deals may be more closely associated with perceived workability (Brzykcy et al., 2019), and may be more instrumental in facilitating recipients’ career development and future performance (Guerrero and Challiol-Jeanblanc, 2016; Liao et al., 2016; Kong et al., 2020). Furthermore, a recent study indicated that flexibility i-deals and reduced workload i-deals may help individuals to focus on other areas in life, such as caring for elderly parents or young baby, and balancing work and family (Bal et al., 2021). Thus, the positive effects of such two types of i-deals may not be visible inside organizations, but may extend beyond organizations. Besides, there usually has a pay or compensation privacy policy in modern organizations, and it is difficult to do compensation research. Therefore, this research explored the influences of development i-deals on recipients’ perceived employability and turnover intention.

### The Retention Path via Perceived Internal Employability

Drawing on social cognitive theory (Bandura, 1986; Wang et al., 2018), this study proposed two paths that linked development i-deals with recipients’ turnover intention via their cognitive processes of perceived employability. The first is a retention path via perceived internal employability, and the second is a turnover path via perceived external employability. In general terms, perceived employability refers to individuals’ evaluations of “capacity to control one’s employment options through the creation, identification, and realization of career opportunities” (Direnzo and Greenhaus, 2011, p. 571). Perceived employability concerns individuals’ perceptions about the possibilities to obtain new employment (De Cuyper et al., 2012). The possibility at their current organization can be referred to as perceived internal employability, and the possibility at another organization can be referred to as perceived external employability (Rothwell and Arnold, 2007; De Cuyper and De Witte, 2010). The difference between them is that the former concerns the transfer of skills within organizational boundaries and the latter outside organizational boundaries.

According to social cognitive theory, Bandura (1978) indicated self-efficacy as a motivational pathway that links contextual factors with individuals’ behaviors. Many scholars have recognized perceived employability as a form of self-efficacy (Nelissen et al., 2017). Given that development i-deals can be viewed as an important contextual factor that can exert significant effects on recipient employees’ self-efficacy (Wang et al., 2018), it is assumed that development i-deals may have a positive influence on their perceived employability. This study speculates that development i-deals can positively impact recipients’ perceived internal employability for the following reasons.

First, as mentioned above, development i-deals refer to employers offering customized opportunities in order to promote particular employees’ working skills and career development, such as on-the-job training, promotion opportunities, or challenging work assignments (Hornung et al., 2009; Zhang et al., 2020). From the perspective of the content, development i-deals may be helpful to increase the recipients’ work ability, and thus enhance their perceived internal employability. Second, the resources that development i-deals involved are usually limited, that is, for example, one employee’s obtaining promotion opportunities may mean that his or her coworkers cannot be raised (Kong et al., 2020). Therefore, granting development i-deals to employees not only means that the recipients can obtain working resources or opportunities that they desire, but also has some invisible implications beyond the actual resources (Srikanth et al., 2020). In other words, authorizing development i-deals delivers a signal that supervisors value and trust the recipients (Rousseau et al., 2006). And obtaining development i-deals can also be viewed as the important evidence of supervisors’ recognition of the i-dealers’ contribution to the organization, their special status in supervisors’ eyes, and supervisors’ expectations of their future performance (Vidyarthi et al., 2016). Those cues from supervisors may cause the recipients’ perception that they are essential for the organization, thus enhancing their assessments of internal employability. Third, development i-deals encourage the recipient employees to participate in their career management (Rousseau et al., 2006). Thus, providing development i-deals may show that the supervisors care about the recipients’ employability. This may strengthen recipients’ beliefs that the employer wants to build a long-term employment relationship with them, which may improve their perception of internal employability. Empirical studies also provide support for this proposition, that individual career management practices could increase their perceptions of internal employability (Soares and Mosquera, 2021). Accordingly, this paper hypothesizes that:

*Hypothesis 1a: Development i-deals are positively related to recipient employees’ perceived internal employability.*

According to social cognitive theory (Bandura, 2006), belief in one’s efficacy is a key personal resource in personal development and change, and such a belief can affect people’s motivation, expectations, and selection. Based on this, this study proposes that perceived internal employability will reduce recipient employees’ turnover intention. Turnover intention is defined as employees’ conscious and deliberate willingness to leave the organization (Tett and Meyer, 1993; Egan et al., 2004). And existing empirical research has shown that increasing an individual’s organizational commitment may be helpful to lower their turnover intention (Kim and Beehr, 2020). Turnover intention and turnover are two distinct concepts, but higher turnover intention is a key element that can predict employees’ turnover behavior (Egan et al., 2004).

Employees who received development i-deals may possess more valuable resources and greater development space than their coworkers who did not obtain i-deals (Huo et al., 2014; Zhang et al., 2020). Those recipients may view leaving the current organization as a risk and a personal loss of resources, and therefore they may not be inclined to leave (De Cuyper and De Witte, 2011). Moreover, based on previous i-deals research, this negative relationship between internal employability and recipient employees’ turnover intention can also be explained through social exchange mechanisms (Liu et al., 2013; Ng and Feldman, 2015; Vidyarthi et al., 2016). Those i-deals studies have shown that when employees successfully negotiate i-deals with their supervisors, they may perceive that their organization values them, trusts them, and provides desired opportunities to facilitate their career development and improve their future performance. As a result, they may feel obliged to respond reciprocally, and thus increase their intention to remain. Furthermore, many scholars have explicitly stated that perceived internal employability can decrease individuals’ intention to leave (Kammeyer-Mueller et al., 2005). A recent study also demonstrated the association between perceived internal employability and lower turnover intention, as well as the mediating role of perceived internal employability in the relationship between career development management and turnover intention (Soares and Mosquera, 2021). Therefore, this paper hypothesizes that:

*Hypothesis 1b: Perceived internal employability is negatively related to recipient employees’ turnover intention.*
*Hypothesis 1c: Perceived internal employability mediates the relationship between development i-deals and turnover intention.*

### The Turnover Path via Perceived External Employability

Similar to perceived internal employability, perceived external employability can also be viewed as a form of self-efficacy (Nelissen et al., 2017). According to the research on employability, employees’ perceived external employability refers to their perception of finding a comparable desired new job with another employer (Rothwell and Arnold, 2007; De Cuyper and De Witte, 2010). Based on the rationale of social cognitive theory (Bandura, 1978), this study posits that development i-deals not only increase the recipient employees’ perceived internal employability, but also increase their external employability at the same time.

First, as noted above, obtaining development i-deals means the supervisors value the recipient employees, that is supervisors recognize the recipients’ contribution to the organization and trust their personal competencies (Rousseau et al., 2006; Vidyarthi et al., 2016). Those clues may improve the recipient employees’ evaluations of their working abilities and self-worth, thus increasing their confidence to find a new job in the external labor market. Second, the within-group heterogeneity nature of development i-deals reveals that development i-deals are not available for every employee, but for someone who is professional or valuable (Marescaux et al., 2019, 2021). Because of this nature, development i-deals may have broader significance and implications beyond the current organization (Bal and Rousseau, 2015). Besides, receiving special work arrangements vis-à-vis career development and skill improvement also shows the recipients’ capabilities and worth to other employers to some degree. In other words, employers can observe the recipient employees’ positive and valuable characteristics, competencies, and attitudes through their experience of obtaining development i-deals. Such experience delivers a powerful signal of the recipient employees’ abilities, worth, and potential to prospective employers (Ho and Kong, 2015). When recipient employees perceive development i-deals as valuable signs of their ability to other employers, the self-perceived external employability may be enhanced. Previous research has provided empirical support for this proposition, that development i-deals could boost recipients’ perception of skill acquisition and occupational self-efficacy (Hornung et al., 2014). Thus, this paper hypothesizes that:

*Hypothesis 2a: Development i-deals are positively related to recipient employees’ perceived external employability.*

According to social cognitive theory, the extent to which individuals believe in their capacity will determine their motivation, affect, and what they decide to do with their abilities, skills, and knowledge (Bandura, 1978, 2006). Bandura (2006) also stated that employees’ cognition about the future could be brought into the present as guides and motivators of current behaviors. Based on these rationales of social cognitive theory, it is speculated that recipient employees’ perceived external employability may increase the risk of their turnover.

Drawing on social cognitive theory, recipient employees with a higher-level perception of external employability may be more likely to feel confident about their working abilities. Such confidence may enhance their self-expectation, which may trigger a belief that they deserve a better organizational platform or a higher position that they desire. Therefore, those recipient employees may be inclined to keep their eyes on job alternatives across organizational boundaries and seek more opportunities outside their own organization, causing their turnover intention to increase. Furthermore, previous research has linked employees’ perceptions of job alternatives to turnover intention, thus providing some support for this proposition. For example, March and Simon (1958) indicated that employees’ perceptions of a number of external alternatives may be an important predictor shaping their turnover intention. Thus, the more alternatives the recipient employees perceive, the more likely they will quit. Many empirical studies have also demonstrated a positive relationship between perceived external employability and turnover intention (Steel and Griffeth, 1989; Hom et al., 1992; Nelissen et al., 2017). Accordingly, this paper hypothesizes that:

*Hypothesis 2b: Perceived external employability is positively related to recipient employees’ turnover intention.*
*Hypothesis 2c: Perceived external employability mediates the relationship between development i-deals and turnover intention.*

### The Moderating Role of Opportunity to Perform

Based on social cognitive theory (Bandura, 1986; Wang et al., 2018), as an important contextual factor, opportunity to perform in current organization may have a significant impact on recipient employees’ cognition, which may alter their reaction toward self-evaluation. The opportunity to perform refers to recipient employees’ perceptions concerning whether they have adequate opportunity to demonstrate their knowledge, skills, and abilities in the current organization (Schleicher et al., 2006; Ingold et al., 2016). And lack of opportunities has long been associated with deviant or counterproductive behaviors (Khan et al., 2017). This study proposes that recipient employees’ perceptions of opportunity to perform in the current organization may moderate the relationships between perceived internal and external employability and turnover intention.

Recipient employees’ perceptions of opportunity to perform emphasize the extent to which they perceive that their current circumstances allow them to express themselves (Schleicher et al., 2006). Recipient employees with a high level of opportunity to perform may perceive that they not only have special opportunities for training or improving their work-related skills, but also the opportunities to develop advanced skills and knowledge to optimize the completion of tasks, thus demonstrating or showing their abilities (Ford et al., 2006). Moreover, the i-deals literature has indicated that i-deals can deliver a signal to the recipient employees that they may have a broader development space than their coworkers (Zhang et al., 2020). And recipient employees’ perceptions of high opportunity to perform may further enhance such cognition. When recipient employees perceive an optimistic picture in the near future, they may be more likely to remain in the current organization, rather than leaving and taking a risk. That is, even though development i-deals can impact recipient employees’ perceptions of their internal and external employability, a high level of opportunity to perform in the current organization may be helpful to increase the recipient employees’ intention to remain. Thus, this study hypothesizes that:

*Hypothesis 3a: Opportunity to perform strengthens the negative effect of perceived internal employability on turnover intention.*
*Hypothesis 3b: Opportunity to perform weakens the positive effect of perceived external employability on turnover intention.*

## Materials and Methods

### Participants and Procedures

The sample covers three companies in Shandong, China. One of them is a high-tech company with about 200 employees, the second one is a traditional textile company with around 800 employees, and the third one is a wholly-owned subsidiary of a large-scale 3C products R&D and manufacturing company, with about 1,500 employees. As a first step, permission was obtained from the CEO of each company to collect the required data. The researchers randomly selected and interviewed a small number of managers and employees from those companies to ensure that development i-deals were a feature of each company. Then the research team acquired lists identifying all participants, and assigned a four-digit code to each of them. With the assistance of the human resource department in each company, all participants were invited into a large conference room. Next, the research team distributed printed questionnaires, gift incentives, and introduced the purpose and procedures of the survey. Participation was voluntary and individuals were assured that their data would remain confidential. After completed the questionnaires, they put them in sealed envelopes and handed them directly to the research team.

To reduce the potential common method bias, the research team collected two waves of data and the interval between each wave was 1 month. At time 1, researchers distributed 410 questionnaires, and asked the participants to report their development i-deals, perceptions of opportunity to perform in the current organization, their organizational commitment, and demographic variables. At time 2, researchers asked them to report their perceived internal and external employability, and their turnover intention. Finally, the research team obtained a complete set of valid responses from 337 individuals (82.20% of the initial sample). Overall, 52.82% were male, and the average age was 36.93. Their average organizational tenure was 8.04 years, and 50.45% held a master’s degree or above. In terms of the position, 29.38% of them were in management positions, 21.96% were designers, 8.90% were financial staff, 28.19% were engaged in research and development, and 11.57% were engaged in product operations.

### Measures

Because all the measures this study used were originally specified in English, back-translation was used to create accurate and understandable Chinese versions (Brislin, 1980). All items were rated on a 7-point Likert-type scale ranging from 1 (*completely disagree or not at all*) to 7 (*completely agree or to a very great extent*).

#### Development I-Deals

Development i-deals were measured with Rousseau and Kim (2006) 4-item scale. The effectiveness of this scale has been confirmed by Wang et al. (2018) and Zhang et al. (2020). And a sample item is “To what extent have you asked for and successfully negotiated with your supervisor for training opportunities” (α = 0.743).

#### Perceived Internal and External Employability

Perceived internal employability was measured with a 4-item scale from De Cuyper and De Witte (2010). This scale has been shown a high reliability in Nelissen and colleagues’ work (2017). A sample item is “I am optimistic that I would find another job with this employer, if I looked for one” (α = 0.819). The items measuring perceived external employability were comparable except for the reference to “another employer” or “elsewhere”. A sample item is “I am optimistic that I would find another job elsewhere, if I looked for one” (α = 0.882).

#### Opportunity to Perform

Opportunity to perform was measured with the Chance to Perform Scale by Bauer et al. (2001). This 4-item scale has been employed by Ingold et al. (2016) in their study to measure employees’ perceptions of opportunity to perform in the current organization. A sample item is “I could really show my skills and abilities in the current organization” (α = 0.901).

#### Turnover Intention

A 4-item scale from O’Reilly et al. (1991) was used to measure turnover intention. This scale’s high reliability has been proved by Ferreira et al. (2017). A sample item is “I prefer another more ideal job than the one I now work in” (α = 0.754).

#### Control Variables

Following Liu et al. (2013) which explored the effects of i-deals on recipient employees, this study controlled for employee demographics, including age, gender, education level, and organizational tenure. In addition, previous research has suggested that employees’ commitment may have a significant direct impact on their turnover intention (Chang, 1999). Thus, this study included recipient employees’ organizational commitment to the current organization as a control variable, using a six-item scale from Meyer et al. (1993). A sample item is “I would be very happy to spend the rest of my career with this organization” (α = 0.905).

## Results

### Common Method Bias Test

Given that all variables in this study were self-reported by recipient employees, Harman’s single factor test was used to examine whether there is a problem of common method bias. The results showed that the variance interpretation rate of the largest factor in the four factors isolated in this study was 26.45%, which was less than 40% of the critical standard. Thus, there was no serious common method bias in this study.

### Confirmatory Factor Analysis

This study analyzed the data using Mplus 7 and SPSS 25. Before testing the hypotheses, a confirmatory factor analysis (CFA) was conducted to examine the validity of the six key variables in the model (development i-deals, perceived internal employability, perceived external employability, turnover intention, organizational commitment, and opportunity to perform in current organization). Chi-square, the Tucker–Lewis index (TLI), the comparative fit index (CFI), the root mean square error of approximation (RMSEA), and the standardized root mean residual (SRMR) were used to assess model fit. As shown in Table 1, the proposed six-factor model showed a good overall fit to the data with χ^2^/df = 1.112, CFI = 0.992, TLI = 0.991, RMSEA = 0.018, SRMR = 0.037, and all factor loadings were significant.

**TABLE 1:** Results of confirmatory factor analysis.

Model	χ^2^	df	CFI	TLI	RMSEA	SRMR
Six-factor model	315.895	284	0.992	0.991	0.018	0.037
Five-factor model-1	574.268	289	0.931	0.922	0.054	0.064
Five-factor model-2	579.488	289	0.930	0.921	0.055	0.065
Five-factor model-3	598.951	289	0.925	0.915	0.056	0.071
Five-factor model-4	605.945	289	0.923	0.914	0.057	0.074
Four-factor model-1	822.938	293	0.871	0.857	0.073	0.080
Four-factor model-2	877.236	293	0.858	0.843	0.077	0.094
Four-factor model-3	995.610	293	0.830	0.811	0.084	0.119
Four-factor model-4	1061.815	293	0.813	0.793	0.088	0.123
Three-factor model-1	1238.557	296	0.771	0.749	0.097	0.128
Three-factor model-2	1767.720	296	0.643	0.608	0.121	0.142
Three-factor model-3	2131.071	296	0.555	0.511	0.136	0.169
Two-factor model-1	2005.381	298	0.586	0.548	0.130	0.149
Two-factor model-2	2373.598	298	0.496	0.451	0.144	0.175
One-factor model	3138.535	299	0.311	0.251	0.168	0.191

*One-factor model = DI + PIE + PEE + TI + OP + OC; two-factor model-1 = DI + PIE + PEE + TI + OP, and OC; two-factor model-2 = DI + PIE + PEE + TI + OC, and OP; three-factor model-1 = DI + PIE + PEE + TI, OP, and OC; three-factor model-2 = DI, PIE + PEE + TI + OP, and OC; three-factor model-3 = DI, PIE + PEE + TI + OC, and OP; four-factor model-1 = DI + PIE + PEE, TI, OP, and OC; four-factor model-2 = DI + TI + OC, PIE, PEE, and OP; four-factor model-3 = DI, PIE + PEE + TI, OP, and OC; four-factor model-4 = DI + TI + OP, PIE, PEE, and OC; five-factor model-1 = DI, PIE + PEE, OP, TI, and OC; five-factor model-2 = DI, PIE, PEE, OP, and TI + OC; five-factor model-3 = DI + OP, PIE, PEE, TI, and OC; five-factor model-4 = DI + TI, PIE, PEE, OP, and OC; six-factor Model = DI, PIE, PEE, TI, OP, and OC. DI, development i-deals; PIE, perceived internal employability; PEE, perceived external employability; TI, turnover intention; OP, opportunity to perform; OC, organizational commitment.*

To determine the discriminant validity of the six constructs, competing models were constructed. As shown in Table 1, the six-factor model fits the data better than other competing models, indicating that the proposed six constructs have good discriminant validity. Therefore, these six constructs were used in the subsequent data analysis.

### Descriptive Analyses

Means, standard deviations, zero-order Pearson correlations, and scale reliabilities for each variable are shown in Table 2. Recipient employees’ development i-deals are positively correlated with their perceived internal employability (*r* = 0.192, *p* < 0.01), and their perceived external employability (*r* = 0.227, *p* < 0.01). Recipient employees’ perceived internal employability is negatively correlated with turnover intention (*r* = −0.344, *p* < 0.01). These results provide preliminary support for the study’s hypotheses.

**TABLE 2:** Means, standard deviations, and correlations.

Variable	*M*	*SD*	1	2	3	4	5	6	7	8	9	10	11
(1) Age	36.93	6.87	–										
(2) Gender^*a*^	0.53	0.50	−0.006	–									
(3) Education^*b*^	2.38	0.69	0.283**	−0.036	–								
(4) Tenure	8.04	4.27	0.675**	−0.030	−0.101	–							
(5) Position^*c*^	2.71	1.44	0.031	0.080	0.062	−0.029	–						
(6) Organizational commitment	4.30	1.09	−0.042	−0.035	−0.011	−0.071	−0.001	*0.905*					
(7) Development i-deals	4.14	0.99	−0.041	−0.093	−0.002	−0.011	−0.024	0.027	*0.743*				
(8) Perceived internal employability	4.82	1.27	−0.041	−0.015	−0.025	−0.018	0.046	0.134*	0.192**	*0.819*			
(9) Perceived external employability	4.41	1.50	−0.001	−0.021	0.017	−0.007	−0.051	−0.026	0.227**	0.617**	*0.882*		
(10) Turnover intention	3.65	1.06	0.026	0.025	0.110*	−0.009	−0.033	−0.512**	−0.129*	−0.344**	0.091	*0.754*	
(11) Opportunity to perform	3.72	1.31	−0.089	−0.017	−0.082	−0.084	−0.078	−0.008	0.150**	0.228**	0.177**	−0.219**	*0.901*

Cronbach’s alphas are shown along the diagonal in italics; ** *p* < 0.01, * *p* < 0.05, the same is true for the tables below. ^*a*^0 = female and 1 = male. ^*b*^1 = associate degree or below, 2 = undergraduate degree, 3 = master degree or above. ^*c*^1 = management positions, 2 = designers, 3 = financial staff, 4 = research and development, 5 = product operations.

Most of the effect sizes of focal variables are in the range of “small” and “medium” sized effects. Specifically, the absolute value of correlations between development i-deals and other focal variables ranges from 0.129 to 0.227, which fits into Rhoades and Eisenberger (2002) interpretation of Cohen (1988) small effect size range (i.e., | *r*| < 0.24). The absolute value of correlations between predictors and turnover intention is higher, ranging from 0.129 to 0.344. Among them, the 0.344 is the medium effect size according to Rhoades and Eisenberger (2002) interpretation of Cohen (1988) medium effect size range (i.e., 0.24 < | *r*| < 0.36), and the rest are the small effect size.

### Hypotheses Testing

To test the proposed hypotheses, the PROCESS macro in SPSS 25 (Hayes, 2013) with a 5,000-resample bootstrap method was used to construct 95% bias-corrected confidence intervals (CIs). If the CI did not include 0, the null hypothesis of no (conditional) indirect effect was rejected (Preacher et al., 2007).

To test direct and indirect effects, PROCESS model 4 was executed. Specifically, in PROCESS model 4, mediators (M) mediate the relationship between the independent variable (*X*) and dependent variable (*Y*). As shown in Table 3, recipient employees’ development i-deals were positively related to perceived internal employability (β = 0.244, 95% CI = [0.124, 0.357]), and recipient employees’ perceived internal employability were negatively related to their turnover intention (β = −0.442, 95% CI = [−0.563, −0.326]). Thus, H1a and H1b were supported. Table 3 also showed that recipient employees’ development i-deals were positively associated with perceived external employability (β = 0.347, 95% CI = [0.218, 0.475]), and recipient employees’ perceived external employability was positively related to their turnover intention (β = 0.305, 95% CI = [0.216, 0.400]). Thus, H2a and H2b were supported.

**TABLE 3:** Bootstrapping results for mediation relationship tests.

Path	Effect	Boot *SE*	Boot LL 95% CI	Boot UL 95% CI
Development i-deals → Perceived internal employability	0.244	0.059	0.124	0.357
Perceived internal employability → Turnover intention	−0.442	0.061	−0.563	−0.326
Development i-deals → Perceived internal employability → Turnover intention	−0.108	0.031	−0.173	−0.051
Development i-deals → Perceived external employability	0.347	0.065	0.218	0.475
Perceived external employability → Turnover intention	0.305	0.047	0.216	0.400
Development i-deals → Perceived external employability → Turnover intention	0.106	0.026	0.060	0.161
Development i-deals → Turnover intention	−0.123	0.044	−0.211	−0.036

*All coefficients are unstandardized. Boot, bootstrapped estimate. SE, standard error. LL, lower level. UL, upper level. CI, confidence interval.*

H1c predicted the mediating role of recipient employees’ perceived internal employability in the relationship between development i-deals and turnover intention, and H2c predicted the mediating role of recipient employees’ perceived external employability in the relationship between development i-deals and turnover intention. Table 3 not only lists the estimates of Stage I effects (independent variable [IV] → mediator [Me]) and Stage II effects (Me → dependent variable [DV]), but also the indirect effects (IV → Me → DV). As hypothesized, perceived internal employability exerted significant mediation effects on the relationship between development i-deals and recipient employees’ turnover intention (indirect effect = −0.108, 95% CI = [−0.173, −0.051]), and perceived external employability also exerted significant mediation effects on the relationship between development i-deals and recipient employees’ turnover intention (indirect effect = 0.106, 95% CI = [0.060, 0.161]). Thus, H1c and H2c were supported.

PROCESS model 14 was executed to test H3a and H3b. Specifically, in this model, one moderator (W) moderates the relationship between the mediators (M_1_, M_2_) and dependent variable (Y). As shown in Table 4, it was revealed that the interaction between perceived internal employability and recipient employees’ perceptions of opportunity to perform in the current organization is negatively related to recipient employees’ turnover intention (β = −0.072, *SE* = 0.034, *p* < 0.01), and the interaction between perceived external employability and recipient employees’ perceptions of opportunity to perform is also negatively related to recipient employees’ turnover intention (β = −0.066, *SE* = 0.030, *p* < 0.01). Thus, recipient employees’ perceptions of opportunity to perform in the current organization strengthened the negative effect of perceived internal employability on turnover intention, and weakened the positive effect of perceived external employability on turnover intention, supporting H3a and H3b.

**TABLE 4:** Moderating effect of opportunity to perform on the relationship between perceived employability and turnover intention.

Variable	Effect	Boot *SE*	Boot LL 95% CI	Boot UL 95% CI
*Y*: Turnover intention				
Constant	5.809	0.361	5.105	6.523
*X*: Development i-deals	−0.107	0.040	−0.186	−0.026
*M*_1_: Perceived internal employability	−0.407	0.061	0.526	−0.290
*M*_2_: Perceived external employability	0.277	0.046	0.188	0.361
*W*: Opportunity to perform	−0.120	0.037	−0.192	−0.044
Interaction 1: *M*_1_ × *W*	−0.072	0.034	−0.138	−0.004
Interaction 2: *M*_2_ × *W*	−0.066	0.030	−0.122	−0.007

**FIGURE 2:**
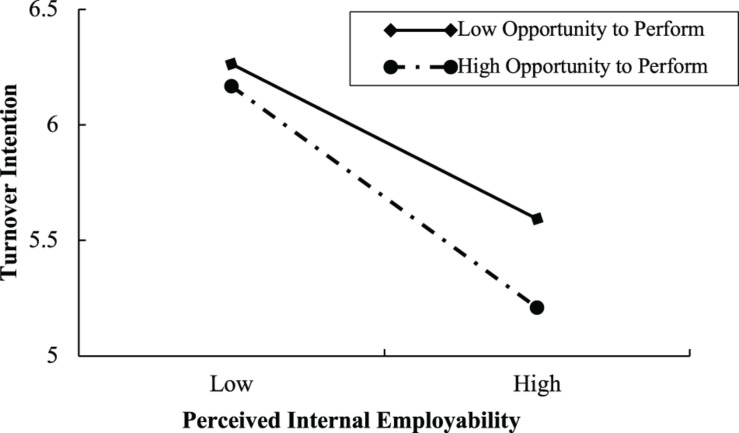
The moderating effect of opportunity to perform on the relationship between perceived internal employability and turnover intention.

**FIGURE 3:**
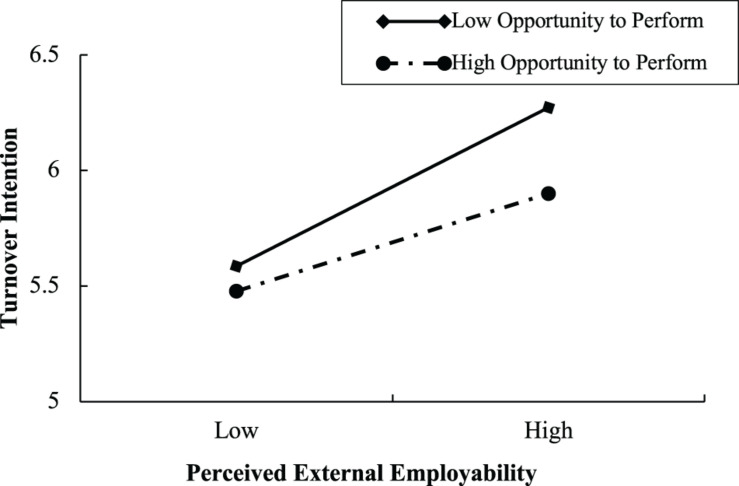
The moderating effect of opportunity to perform on the relationship between perceived external employability and turnover intention.

To further test the moderating effect, this study conducted a simple slope analysis (Aiken et al., 1991), demarcating between high (one standard deviation above the mean) and low (one standard deviation below the mean) levels of recipient employees’ perceptions of opportunity to perform in the current organization. As shown in Figure 2, the negative influence of perceived internal employability on recipient employees’ turnover intention is weaker for recipient employees with lower perceptions of opportunity to perform than those with higher perceptions of opportunity to perform. Figure 3 shows that the positive influence of perceived external employability on recipient employees’ turnover intention is weaker for recipient employees with higher perceptions of opportunity to perform in the current organization than those with lower such perceptions.

## Discussion

Drawing on social cognitive theory, the current research proposed and tested a moderated mediation model to explain the mechanisms through which development i-deals affect recipient employees’ turnover intention. Specifically, this study proposed two paths that linked development i-deals with turnover intention: a retention path via perceived internal employability and a turnover path via perceived external employability. Through a time-lagged research design, the results supported proposed hypotheses, showing that the effects of development i-deals on recipients’ turnover intention may be complex. The results found that development i-deals can improve recipients’ perception of internal employability, which is negatively related to their turnover intention. This finding is consistent with previous i-deals studies which hold the argument that customized development work arrangements are associated with lower turnover intention (Hornung et al., 2014; Liao et al., 2016). And this finding is also in accordance with the work of Hornung et al. (2010), showing that development i-deals can increase recipient employee’s work engagement. Besides, different from traditional assumptions, the results also found that development i-deals can enhance recipients’ perception of external employability, which is positively related to turnover intention. This finding is parallel with that of Rodrigues et al. (2020), who suggested that individuals can benefit from organizational investment in their career development practices, such as feel highly employable, and their perceived external employability is positively associated with intention to quit. Furthermore, the results confirmed the moderating effects of opportunity to perform in current organization on the relationships between perceived employability and turnover intention. This finding supports literature suggesting that a positive perception of the availability of career opportunities within one’s organization is critical to retaining employees (Rasheeda et al., 2020).

### Theoretical Implications

This study has several theoretical implications. First, it contributes to i-deals literature by building a model to reveal the process through which development i-deals impact recipients’ turnover intention. Previous studies indicated that development i-deals satisfy the i-dealers’ personal needs, thus increasing their commitment and willingness to remain (Liu et al., 2013; Las Heras et al., 2017). Consistent with previous studies, this research proposed a retention path and the results found that development i-deals can improve recipients’ perceived internal employability, which in turn reduce their turnover intention. Besides, this paper also verified a turnover path that development i-deals can increase i-dealers’ perceived external employability and thus the risk of turnover. This study responds to the call proposed by Ng (2017) that more research should be conducted to explore the potential negative effects of granting i-deals. Thus, this study extends i-deals literature by investigating the effect of development i-deals on recipients’ turnover intention, revealing the benefits and risks that development i-deals may bring to the organization.

Second, this study contributes to social cognitive theory by examining the mediating roles of recipients’ cognitive processes which can be conceptualized as the employability paradox in the relationship between development i-deals and turnover intention. Previous research on i-deals is dominated by mediating mechanisms pertaining to social exchange, social identity, or social comparison perspectives (Ng and Feldman, 2015; Vidyarthi et al., 2016; Zhang et al., 2020). However, this study tries to explore how receiving development i-deals shapes the i-dealers’ self-cognition, and thus their turnover intention. This research adds the limited mediating mechanisms linking development i-deals with recipients’ behaviors from a social cognitive perspective. Thus, it responds to scholars’ calls (Bal and Rousseau, 2015; Liao et al., 2016) that more research should drill down into the mechanisms through which i-deals have an impact on outcomes from different theoretical perspectives, and it also enriches social cognitive theory by applying this theory into the i-deals context.

Third, this study increases the understanding of human resource development by investigating the relationship between development i-deals and the recipients’ perceived employability. That is, granting individualized work terms about career development to employees can boost both their perceptions of internal and external employability. This study explains the phenomenon that why some employees remain and why some leave after they were given development i-deals, providing theoretical support for this employability paradox. Besides, this study also examined the boundary effects of opportunity to perform in current organization. And it responds to the suggestion of Liao et al. (2016) that greater consideration was needed of contextual moderators in research on i-deals. The results showed that for recipients who perceive more opportunity to perform in current organization, the positive relationship between perceived external employability and turnover intention can be weaker. This finding provides some theoretical evidence about how to avoid the potential turnover risks and how to maximize the effectiveness of development i-deals.

### Practical Implications

This study has some practical implications for managers and organizations. First, the results of this research showed that development i-deals can increase recipient employees’ perceptions of internal employability, and subsequently enhance their willingness to remain. This finding revealed the benefits that development i-deals bring for the organization, and verified the original assumption that i-deals can be used to help retain valued employees. Therefore, for those organizations focusing on employee retention, development i-deals can be an instrumental tool to achieve such aims. For instance, managers can provide individualized work arrangements for key employees who have higher working skills, core technologies, professional knowledge, or higher competitiveness. This may be a win-win strategy for employees and managers, that is employees can obtain customized work items and managers retain the talents.

Yet, i-deals have negative ramifications too. Challenging traditional wisdom, it was found that development i-deals can also increase recipient employees’ evaluations of their external employability, and thus increase the risk of turnover intention. This finding revealed the potential cost of providing employees with development i-deals. Thus, when authorizing i-deals, managers should undertake a balanced analysis of benefits and risks. For example, managers could sign a supplementary and reciprocal contract about i-deals with recipients, which specify obligations and duties of the both parties. More specifically, both parties could negotiate the service period before employees are given the special training opportunities, and once they are in violation of the service period stipulation, he or she shall pay the organization a penalty for breach of contract as stipulated. Besides, when authorizing i-deals, managers also should be cautious about the individual difference of i-dealers, such as organizational commitment, loyalty, or identification.

Moreover, the results verified the moderating effect of opportunity to perform in current organization, and showed that the opportunity to perform can alter the effect of perceived employability on recipient employees’ turnover intention. Overall, it is demonstrated that employees with a perception of high level of opportunity to perform are more likely to show greater willingness to remain in the current organization. This finding suggests that in order to maximize the effectiveness of development i-deals, managers and organizations should create more opportunities for i-deals recipients. For example, after the special training, managers should be aware of providing some practice opportunities for those recipient employees to apply the knowledge or skills that they learned from the training courses. Managers also should pay attention to the recipients’ psychological and developmental needs. For instance, managers could help the recipients make a career development plan, in order to reduce their uncertainties, increase their confidence about their future career development in the current organization, and enhance their willingness to build a long-term employment relationship with current employer.

### Limitations and Future Research

This research may have some limitations. The first is that all variables in the model were assessed with self-reported responses. This paper addressed recipient employees’ receiving development i-deals and their psychological cognitive process, so self-reporting of these variables was not unreasonable, even though this may raise the possibility of common method bias (Podsakoff et al., 2003). Indeed, it may be difficult to argue that others could provide an accurate evaluation of recipient employees’ subjective feelings. This study tried to minimize the potential influence of common method bias on the results by separating the data collection into two measurement waves and randomizing the order of scale items in the survey instrument.

Second, this study was conducted only in Shandong, China, which may limit the generalizability of the results in some degree. Future studies could reexamine whether development i-deals may influence recipient employees’ turnover intention via employability paradox, in other areas of China or other country settings. Besides, this research examined the effect of development i-deals on recipients’ turnover intention, revealing the possible risk that development i-deals bring for the organizations. Future research is needed to explore whether and in which conditions, development i-deals can lead to recipients’ actual turnover behaviors by utilizing a longitudinal tracing design. Moreover, this study only focused on development i-deals, and future research could investigate the influence of other types of i-deals on the recipients’ perceived employability and turnover intention, and compare such influence with that of development i-deals.

Third, this study used social cognitive theory to introduce the recipients’ perceived employability paradox as the theoretically-driven mediators that linked development i-deals and turnover intention. However, there may exist other theoretical frameworks that can be used to understand the employability paradox. For example, future research could analyze the employability paradox by using social exchange theory. In addition, many scholars have indicated that i-deals have broader implications beyond the recipient employees (Marescaux et al., 2021). Thus, it is encouraged that future research could explore the impacts of authorizing i-deals on the i-dealers’ coworkers or supervisors themselves.

## Conclusion

Drawing on social cognitive theory, this study revealed the mechanism of the mediating effects of the employability paradox between development i-deals and recipient employee’s turnover intention. This study also found that recipient’s perception of opportunity to perform in current organization plays a moderating role. The results advance collective understandings of how development i-deals impact recipient’s turnover intention, providing insights that could be leveraged by human resource personnel.

## Data Availability Statement

The original contributions generated for this study are included in the article/supplementary material, further inquiries can be directed to the corresponding authors.

## Ethics Statement

The studies involving human participants were reviewed and approved by the Ethical Review Board of Beijing Jiaotong University. The patients/participants provided their written informed consent to participate in this study.

## Author Contributions

XZ contributed to the design of the research model and the preparation of the manuscript. HD contributed to the analysis and interpretation of the data. YX contributed to the collection of the data. YL contributed to the revision of the manuscript. All authors contributed to the article and approved the submitted version.

## Conflict of Interest

The authors declare that the research was conducted in the absence of any commercial or financial relationships that could be construed as a potential conflict of interest.

## Publisher’s Note

All claims expressed in this article are solely those of the authors and do not necessarily represent those of their affiliated organizations, or those of the publisher, the editors and the reviewers. Any product that may be evaluated in this article, or claim that may be made by its manufacturer, is not guaranteed or endorsed by the publisher.
